# Youth Psychopathic Traits Inventory-Short Version: A Further Test of the Internal Consistency and Criterion Validity

**DOI:** 10.1007/s10862-012-9299-0

**Published:** 2012-06-26

**Authors:** Olivier F. Colins, Marc Noom, Wouter Vanderplasschen

**Affiliations:** 1 Department of Child and Adolescent Psychiatry, Curium-LUMC/Leiden University Medical Center, Leiden, The Netherlands; 2 Department of Special Education, Ghent University, Ghent, Belgium; 3 Endergeesterstraatweg 27, 2342 AK Oegstgeest/Leiden, The Netherlands

**Keywords:** Antisocial, Assessment, Self-report, Psychopathy

## Abstract

The Youth Psychopathic Traits Inventory-Short Version (YPI-S; van Baardewijk et al., 2010) is a self-report measure to assess psychopathic-like traits in adolescents. The aim of the present study is to investigate the factor structure, the internal consistency, and the criterion validity of the YPI-S in 768 Belgian community adolescents (45.4 % males). In general, our study supported the YPI three factor structure while relevant indices showed that the instrument is internally consistent. In addition, relations between the YPI-S total score and dimension scores on the one hand and external criterion measures (e.g. conduct problems and self-reported offending) on the other hand were generally in line with predictions. The present study replicated and substantially extended previous findings of the YPI-S in a sample of community youth. Future studies are needed to test whether findings from community samples can be replicated in clinical-referred and justice-involved boys and adolescents.

## Introduction

The constellation of interpersonal, affective and behavioral traits referred to as psychopathy has proven to be important to identify serious and violent antisocial adults. Because an individual does not suddenly become a psychopath at the moment one turns 18 years of age (e.g. Salekin et al. 2004), many researchers tried to identify early manifestations of psychopathy in childhood and adolescence. Several instruments have been developed to measure psychopathic-like traits in minors. Although the Psychopathy Checklist: Youth Version (PCL:YV; Forth et al. 2003) is often considered to be the most reliable and valid measure of psychopathic-like traits among forensic youth (Andershed et al. 2007), it is also the most expensive and time-consuming. Furthermore, the PCL:YV’s reliance on file-information makes this instrument difficult to use in settings where file data is absent (e.g. community samples). In such settings, researchers most often use alternative screening measures that rely on parents, teachers and/or youths themselves. When compared with instruments relying on parent and teacher ratings, youth self-report instruments are relatively new and thus less studied.

Using self-report instruments to examine psychopathic-like traits in adolescents is important for several reasons. First, adolescents have the opportunity to report on behaviors, emotions and thoughts across a range of situations. Thus, self-report may be a relevant source of information when unraveling the etiology of psychopathy (Munoz and Frick 2007). Second, using self-report assessment of psychopathic-like traits seems particularly relevant when parents or teachers are not available or when they did not have enough contact with their child to provide useful information on the presence or absence of these personality traits (Loney et al. 2003). Third, a self-report questionnaire is easy to complete and requires minimal training on the part of the test administrator (Lilienfeld and Fowler 2006). This economic advantage makes self-report questionnaires appealing for use in settings that do not have enough financial resources to use expert-based assessment for all youth (e.g. detention centers).

Various self-report instruments are currently available to assess psychopathic-like traits in adolescents (Kotler and McMahon 2010). One instrument that has been considered as promising (e.g. Kotler and McMahon 2010; Skeem and Cauffman 2003) is the Youth Psychopathic Traits Inventory (YPI; Andershed et al. 2002). Based on Cooke and Michie’s model of psychopathy (Cooke and Michie 2001) the YPI assesses ten core psychopathy traits through ten YPI subscales (with five items each) organized in three factors. The psychometric properties of the YPI have generally been described as satisfying. In specific, the YPI seems to have a stable three-factor structure (e.g. Declercq et al. 2009; Larsson et al. 2007a), while also good to excellent internal consistencies have been reported for the total YPI score, the three YPI dimensions and most YPI subscales (e.g. Skeem and Cauffman 2003). Yet, a new and short version of the YPI has been developed through a stepwise selection process using a series of exploratory factor analyses and content related arguments. The final model with 18 items (organized in three factors but without subscales) was then cross-validated in independent samples using confirmatory factor analyses and external validity was tested and compared with the original YPI measure (van Baardewijk et al. 2010).

On the one hand, this item reduction is surprising since a limitation of other self-report instruments is the restricted number of items that capture the interpersonal, affective and behavioral traits of the psychopathy construct (Kotler and McMahon 2010). Therefore, it can be doubted whether the YPI-Short Version (YPI-S) will contribute to our understanding of psychopathic-like traits above and beyond other available self-report instruments. On the other hand, by not including subscales in the YPI-S a concern that relates to an inappropriate use of parceling in factor analyses (FA) may indirectly have been resolved. In all YPI studies, FA were conducted on the ten subscales scores and not on the 50 items (e.g. Andershed et al. 2002; Nijhof et al. 2011). By using subscale scores rather than items, parcels were introduced in the FA. Parceling is a procedure where items are summed to form composite scores prior to FA. Parceling is justified if the assumption of unidimensionality is met, that is, if items in a particular subscale do not load substantially on other subscales (Hart et al. 2007; Little et al. 2002). However, while constructing the YPI and thus, when assigning items to subscales, the assumption of unidimensionality was not examined. From this perspective, the YPI-S may be welcomed as its factor structure may be more straightforward and therefore may help us to increase our understanding of psychopathy (Hart et al. 2007).

In the one YPI-S study published to date (van Baardewijk et al. 2010), the theoretically driven three-factor structure of the YPI-S had good fit indices, while the YPI-S dimensions were found to have moderate to good internal consistency (Cronbach’s alphas of .69 to .71). In addition, the YPI-S total score and YPI-S dimension scores were related with conduct problems in adolescents (correlations of .38 to .42). Despite these promising results there are three major reasons why the internal consistency and validity of the YPI-S should further be examined. First, all YPI-S data in the van Baardewijk et al. (2010) study stems from an administration of the original 50-item YPI. Therefore, it is unclear how the YPI-S performs when participants fill out only the 18 YPI-S items without the remaining 32 original YPI items. Second, revealing a relation between the YPI-S and conduct problems is important albeit not sufficient to demonstrate the criterion validity of the YPI-S. That is, psychopathic-like traits in adolescents are not only related to conduct problems but for example, also positively related to hyperactive behavior (e.g. Sevecke et al. 2009), problems with peers (e.g. Munoz et al. 2008) and criminal behavior (e.g. Poythress et al. 2006). Third, the original YPI-S study (van Baardewijk et al. 2010) used a variable-oriented approach to examine the relation between YPI-S dimensions and criterion measures. Hence, an important question that remains unanswered is whether the YPI-S could identify a particular subgroup of adolescents that exhibited a pattern of high scores on all three YPI-S dimensions, and to what extent this psychopathic-like subgroup, for example, is more delinquent than non-psychopathic-like adolescents (e.g. Andershed et al. 2002).

For reasons mentioned above, the current study was designed to examine (i) whether the three-factor structure of a new self-report questionnaire, the YPI-S, can be replicated in a sample of 768 Belgian community adolescents; (ii) to examine the internal consistencies of the YPI-S in general and each of its three dimensions in particular; and (iii) to examine the relation between the YPI-S total score/YPI-S factor scores and relevant criterion variables. We hypothesized that the YPI-S total score would be positively related with self-reported offending, conduct problems, hyperactivity and problems with peers. With regard to the YPI-S dimension scores, and based on previous studies on psychopathic-like traits in adolescents (e.g. Poythress et al. 2006), we specifically expected that the behavioral dimension of the YPI-S is most strongly related to conduct problems and hyperactivity, while only the affective dimension is negatively related to emotional problems. Finally, we hypothesized that a subgroup of youths scoring high on all three YPI-S dimensions (i.e. psychopathic-like juveniles) will report more conduct problems and offenses than their non-psychopathic-like counterparts.

## Method

### Participants

Data was collected in 7 secondary schools for general, technical, vocational and/or art education in the region of Ghent (Belgium) between February 2010 and May 2010. Of the 860 eligible participants that we could approach (i.e. target sample), 51 were absent on the day of the data collection and 11 parents did not gave consent. None of the juveniles themselves refused to participate, resulting in a sample size of 798 youth who filled out the questionnaires. Seven juveniles were excluded from the study because of insufficient Dutch language skills, resulting in a sample size of 791 participants. YPI-S data (ranging from 1 to 18 items) was missing for 76 participants. To include as many cases as possible, missing values were imputed using the missing values analysis procedure in SPSS 18.0, with the expectation maximization method, resulting in a sample size of 784. In addition, 16 participants were excluded because they were younger than 12 years or were 19 year or older, resulting in final total sample of 768 participants.

Participants (*n* = 768) ranged in age from 12.07 to 18.94 years (mean = 14.85; SD = 1.93). With regard to racial distribution, 90.0 % of the participants were from Belgian origin, while the remaining participants were from Moroccan (1.6 %), Turkish (3.9 %), Dutch (i.e. the Netherlands; 1.2 %) or another origin (3.4 %). This racial distribution is similar to that of the general population in Ghent (i.e. 90 % citizens with Belgian nationality and Turkish, Moroccan and Dutch among the most prevalent non-Belgian nationalities). With regard to type of education 70.4 % attended general schools, 13.2 % art schools, 11.1 % technical schools and 5.3 % vocational schools.

### Procedure

Survey approval was provided by the boards of each school. Students and their parents were informed about the survey administration. All students were surveyed unless they declined to participate or their parents had objections. Administration of the survey was conducted in the classroom on a regular school day. Before starting the assessment, the students were informed again about the confidentiality of the information and signed consent forms. They were asked to complete the questionnaires in their classroom during a one-hour session under the supervision of a specially trained research assistant (master-level student). Students could ask the supervisor for clarification if they did not understand the question. After the students finished their questionnaires, they brought them to the class box that was sealed by the research assistant.

### Measures

#### Youth Psychopathic Traits Inventory-Short Version (YPI-S)

The YPI-S is an 18-item self-report questionnaire designed to measure psychopathic-like traits in adolescents (van Baardewijk et al. 2010). In line with the three factor model of psychopathy (Cooke and Michie 2001), the YPI-S items comprise three factors or dimensions with six items. The Grandiose-Manipulative or interpersonal dimension comprises dishonest charm, manipulation/lying, and grandiosity. The Callous-Unemotional or affective dimension comprises callousness, unemotionality, and remorselessness, and the Impulsive-Irresponsible behavior or behavioral dimension features impulsivity, irresponsible behavior, and thrill-seeking/proneness to boredom. Table 2 displays the items for each dimension. Each item in the YPI is scored on a 4-point Likert scale ranging from “Does not apply at all” to “Applies very well”.

#### The Strengths and Difficulties Questionnaire: Self-Report Version (SDQ)

The SDQ is a brief screening instrument for psychosocial functioning of children and adolescents (Dutch translation: van Widenfelt et al. 2003). The SDQ has five subscales scores. Each subscale consists of 5 items with three response categories (not true = 0, somewhat true = 1, certainly true = 2). With regard to the four difficulty subscales (i.e. hyperactivity, conduct problems, peer problems, emotional symptoms), a higher score refers to a higher level of problem severity. With regard to the strength subscale (i.e. prosocial behavior) a higher score refers to more prosocial behavior. For the SDQ subscales Cronbach’s alpha was 0.70 for emotional problems; 0.51 for conduct problems; 0.71 for hyperactivity; 0.41 for peer problems and 0.63 for prosocial behavior. These Cronbach’s alpha’s were higher than those reported in the validation study of the Dutch version of the SDQ (van Widenfelt et al. 2003).

#### Self-Reported Offending

We used the 36-item WODC Monitor (van der Laan and Blom 2005) to investigate whether participants ever committed an offense from a list of 36 offenses that are targeted. For the current study we used 33 offenses (i.e. items) to create five continuous offense categories. First, seven items referring to violence were classified as “violent offenses”(i.e. using violence to steal, hitting or kicking but not injuring someone, fighting because the other is homosexual, fighting because the other has another skin or color, hitting or kicking with injuries, injuring someone with a weapon, trying to have sex while the other refuses). Second, 16 items referring to income-related non-violent delinquent behavior or deliberately damaging property were classified as “property offenses” (e.g. selling stolen property, burglary, shoplifting, car theft, bike/scooter theft, car damaging, graffiti). Third, three items referring to dealing or selling drugs were classified as “drug-related offenses” (i.e. selling XTC, amphetamine or mushrooms; selling marijuana; selling coke or heroin). Fourth, five items referring to threatening and insulting were classified as “threats and insults” (i.e. threatening to scare someone; threatening to steal from someone; insulting someone because he/she is homosexual, insulting someone because he/she has another skin or color; making someone scare through email, SMS or chat). Finally, two items that could not be assigned to one of the four previous types of offending were classified as “other offending” (i.e. possession of weapons to protect oneself, deliberately sending viruses by email or the internet). The three remaining questions referring to illegal internet downloading, not having a valid ticket for public transport and enlightening firework were not included because these behaviors are very common among adolescents (JOP 2007) or not illegal in Belgium (cf. firework) and may therefore artificially increase the number of participants involved in ‘delinquent’ behavior.

#### Socio-Demographics

Standardized information about age and origin was assessed by means of a self-report questionnaire designed by the authors. Age was dichotomized to testing whether the factor structure of the YPI-S differs between younger and older adolescents. Participants were classified as “younger adolescents (i.e. 12.00 to 14.99 years of age) or “older adolescents” (15.00 to 18.99 years of age).

## Results

### Descriptives

Descriptive information for the total sample, for age groups and for boys and girls is presented in Table 1. One-way analyses of variance demonstrated differences in variables of interest between males and females and between younger and older adolescents. Because a high number of analyses were performed we used an alpha of 0.01 as the standard for statistical significance in these and all other analyses. Cohen’s d was used as measure of the magnitude of the observed effect (i.e. mean group differences), with an effect size of .20 referring to a small effect, .50 to a medium effect, and .80 to a large effect. Comparing boys with girls, medium effect sizes (.47 to .60) were revealed for the total YPI-S score, the interpersonal dimension, the affective dimension, emotional problems and prosocial behavior, with girls only scoring higher on the latter two variables (Table 1). When comparing younger to older adolescents, a large effect size was found for conduct problems (.78), with younger adolescents having more conduct problems than their older counterparts (Table 1).

**Table 1:** Mean (M) and Standard Deviations (SD) for variables of interest

	Total sample (*n* = 768)	Gender				Age group			
		Boys (*n* = 349)	Girls (*n* = 419)	Group comparison	ES	Young (*n* = 431)	Old (*n* = 337)	Group comparison	ES
YPI-S	Mean (SD)	Mean (SD)	Mean (SD)	p		Mean (SD)	Mean (SD)	p	
Total YPI-S score	32.62 (7.14)	34.89 (7.53)	30.77 (6.22)	<0.001	0.60	32.01 (6.90)	33.46 (7.37)	<0.01	0.20
Interpersonal dimension	10.82 (3.60)	11.86 (3.79)	9.95 (3.19)	<0.001	0.55	10.27 (3.34)	11.52 (3.80)	<0.001	0.35
Affective dimension	9.52 (3.04)	10.52 (3.26)	8.69 (2.57)	<0.001	0.63	9.75 (3.00)	9.22 (3.08)	0.02	0.17
Behavioral dimension	12.31 (3.09)	12.51 (3.15)	12.14 (3.04)	0.09	0.12	11.98 (3.00)	12.72 (3.17)	<0.01	0.24
SDQ									
Emotional problems	3.32 (2.34)	2.61 (1.96)	3.92 (2.46)	<0.001	0.59	2.92 (2.20)	3.84 (2.42)	<0.001	0.40
Conduct problems	1.97 (1.55)	2.26 (1.56)	1.65 (1.48)	<0.001	0.40	2.02 (1.61)	1.90 (1.48)	0.28	0.78
Hyperactivity	4.51 (2.30)	4.69 (2.30)	4.35 (2.28)	0.04	0.15	4.28 (2.22)	4.80 (2.36)	<0.01	0.23
Peer problems	1.69 (1.51)	1.74 (1.52)	1.65 (1.48)	0.37	0.06	1.62 (1.53)	1.78 (1.46)	0.16	0.11
Prosocial behavior	7.98 (1.66)	7.56 (1.70)	8.32 (1.55)	<0.001	0.47	7.95 (1.66)	8.00 (1.67)	0.65	0.03
Type of offenses									
Violent offenses	0.54 (0.76)	0.77 (0.89)	0.34 (0.58)	<0.001	0.57	0.45 (0.71)	0.65 (0.81)	<0.001	0.26
Property offenses	1.39 (2.12)	1.74 (2.50)	1.09 (1.68)	<0.001	0.31	0.78 (1.58)	2.16 (2.45)	<0.001	0.67
Drug-related offenses	0.10 (0.37)	0.13 (0.43)	0.07 (0.30)	0.02	0.16	0.02 (0.13)	0.20 (0.51)	<0.001	0.48
Threats and insults	0.41 (0.75)	0.63 (0.88)	0.23 (0.55)	<0.001	0.55	0.32 (0.64)	0.53 (0.85)	<0.001	0.28
Other offenses	0.13 (0.36)	0.21 (0.44)	0.07 (0.27)	<0.001	0.38	0.11 (0.33)	0.17 (0.40)	0.02	0.16

ES = Effect Size (Cohen’s d); YPI-S = Youth Psychopathic Traits Inventory Short Version; SDQ = Strengths and Difficulties Questionnaire; Because YPI-S variables were tested against many criterion variables we used an alpha of 0.01 as the standard for statistical significance.

### Factor Structure

A confirmatory factor analysis (CFA) was performed to test whether the YPI three factor structure can be replicated in our sample. The CFA was performed using LISREL 8.80 (Jöreskog and Sörbom 2006). In line with previous YPI studies that used LISREL (e.g. Andershed et al. 2002; Larsson et al. 2007b) the model fit was estimated using the weighted least squares (WLS) method based on correlation matrices. This method does not assume that the variables (i.e. YPI-S scores) follow a multivariate normal distribution in the population (e.g.Verona et al. 2005). In line with the YPI-S study (van Baardewijk et al. 2010) we calculated the root mean square error of approximation (RMSEA) and the comparative fit index (CFI) and used the following cut-off values to evaluate the model fit: a CFI of .90 and higher, and an RMSEA of .08 and lower are indications of an adequate fit, while a CFI of .95 and higher and an RMSEA of .05 and lower are indications of a good fit (but see: Marsh et al. 2004 for a comment on these commonly applied cut-off values for assessing model fit). To present an straightforward test of the predicted model), we did not add correlated residuals CFA results indicate an adequate fit of the YPI-S’s three factor model for the total sample (χ² = 652.69, df = 132, *p* < .01; CFI = 0.90, RMSEA = 0.072). Multi-group analyses did not reveal any differences in the structural model for gender or age groups (details available upon request). The three factor model with standardized parameter estimates for the total sample is displayed in Table 2. Pearson correlations showed that all three YPI dimensions were significantly albeit modestly correlated with each other, while all YPI dimension scores were highly and significantly correlated with the YPI total score (Table 3). These results confirmed the proposed three factor model for the YPI-S. However, the item “I have probably skipped school or work more than most other people”, shows a relatively low loading of .11. For reasons mentioned in the discussion, we simply wanted to test whether the original YPI-S factor structure could be replicated. Therefore, we did not rerun the CFA after removing this item. To allow other researchers to specify alternative models for these data, we included the correlation matrix and the means/SDs for the 18 items in Appendix 1.

**Table 2:** Standardized factor loadings for the three-factor-model of the Youth Psychopathy Traits Inventory-Short Version (*n* = 768)

Interpersonal dimension	ID	AD	BD
I have the ability to con people by using my charm and smile (4)	.64		
I am good at getting people to believe me when I make something up (5)	.61		
I have talents that go far beyond other people’s (8)	.34		
It’s easy for me to manipulate people (9)	.64		
When I need to, I use my smile and my charm to use others (14)	.59		
I am destined to become a well-known, important and influential person (16)	.31		
Affective dimension			
I think that crying is a sign of weakness even if no one sees you (3)		.53	
When other people have problems. it is often their own fault. therefore one should not help them (6)		.27	
To be nervous and worried is a sign of weakness (10)		.42	
I don’t understand how people can be touched enough to cry by watching things on TV or movie (15)		.49	
To feel guilty and remorseful about things you have done that have hurt other people is a sign of weakness (17)		.38	
I don’t let my feelings affect me as much as other people’s feelings seem to affect them (18)		.39	
Behavioral dimension			
I have probably skipped school or work more than most other people (1)			.11
I consider myself as a pretty impulsive person (2)			.40
It often happens that I talk first and think later (7)			.68
I get bored quickly by doing the same thing over and over (11)			.30
It often happens that I do things without thinking ahead (12)			.80
It has happened several times that I’ve borrowed something and then lost it (13)			.30

ID = interpersonal dimension; AD = affective dimension; BD = behavioral dimension; the latent factor correlation for ID and AD was 0.40 (*p* = 0.04), for ID and BD 0.37 (*p* = 0.04), and for AD and BD 0.15 (*p* = 0.05).

**Table 3:** Pearson correlations between YPI-S factor scores and between YPI-S total score and factor scores and internal consistency indices for YPI-S total score and YPI-S factor scores (total sample)

	Correlations					
	YPI-S total	ID	AD	Alpha	MCITC	MIC
Total YPI-S score	1.00	–	–	0.78	0.35	0.28
Interpersonal dimension (ID)	0.81^***^	1.00	–	0.76	0.50	0.35
Affective dimension (AD)	0.68^***^	0.35^***^	1.00	0.66	0.40	0.25
Behavioral dimension	0.69^***^	0.36^***^	0.18^***^	0.66	0.39	0.24

YPI-S = Youth Psychopathic traits Inventory Short Version ;Alpha = Cronbach’s alpha; MCITC = mean corrected item-to-total correlation; MIC = mean inter-item correlation; *** *p* < 0.001

### Internal Consistency

To evaluate the internal consistency of the YPI-S Cronbach’s alphas are presented. By convention an alpha higher than .70 is indicative of adequate internal consistency. The mean corrected item-to-total correlation (MCITC) helps to identify items that, if excluded from the scale, would improve alpha for that scale, while the mean inter-item correlation (MIC) is considered to be a more straightforward indicator of the internal consistency of a scale than alpha (Clarck and Watson 1995). Besides alphas, we therefore also present MCITC and MIC values. The MCITC should be above the conventionally recommended value of .30 (Nunnally and Bernstein 1994) while MIC values in the range of .15 to .50 are considered adequate (Clarck and Watson 1995). Alphas for the affective dimension (AD) and the behavioral dimension (BD) were slightly below the recommended criterion value of .70. However, for all YPI-S scores the MCITC and the MIC were above the recommended criterion value of .30 and between .15 and .50 respectively (Table 3).

### Criterion Validity: Variable Oriented Approach

#### Unadjusted and Adjusted Models

To validate the YPI-S to criteria relevant for the psychopathy concept, standardized regression coefficients (ß) were calculated to examine the relation between the YPI-S total score or the three YPI dimensions and criterion measures (i.e. unadjusted models). For YPI-S dimensions, ß were also calculated to examine the relation between each YPI-S dimension and criterion measures after adjusting for the other two YPI-S dimensions (i.e. adjusted model). The total YPI-S score was unrelated with emotional problems, and positively related with conduct problems, hyperactivity, problems with peers and all types of offenses (Table 4). The adjusted models presented in Table 4 shows that the interpersonal dimension (ID) was positively related with conduct problems and all types of offenses. The AD was positively associated with conduct problems, peer problems, violent offenses, threats and insults and other offenses, and negatively with emotional problems. The BD was positively related with almost all criterion variables.

**Table 4:** Unadjusted and adjusted standardized regression coefficients to examine the relation between YPI-S dimensions (standardized) and criterion variables (total sample)

SDQ	YPI-S total	Interpersonal dimension		Affective dimension		Behavioral dimension	
	Unadjusted ß	unadjusted ß	(adjusted ß)	Unadjusted ß	(adjusted ß)	Unadjusted ß	(adjusted ß)
Emotional problems	−0.02	−0.02	(−0.10)	−0.13***	(−0.15***)	0.10**	(0.13**)
Conduct problems	0.43***	0.33***	(0.18***)	0.26***	(0.15***)	0.37***	(0.28***)
Hyperactivity	0.34***	0.21***	(0.05)	0.05	(−0.06)	0.50***	(0.50***)
Peer problems	0.13***	0.11**	(0.07)	0.14***	0.12**	0.04	(−0.01)
Prosocial behavior	−0.37***	−0.27***	(−0.14***)	−0.31***	(−0.23***)	−0.23***	(−0.14***)
Types of Offense							
Violent offenses	0.38***	0.34***	(0.25***)	0.27***	(0.16***)	0.18***	(0.06)
Property offenses	0.41***	0.40***	(0.32***)	0.17***	(0.02)	0.29***	(0.17***)
Drug-related offenses	0.26***	0.27***	(0.24***)	0.08	(−0.03)	0.20***	(0.11**)
Threats and insults	0.44***	0.42***	(0.33**)	0.31***	(0.18***)	0.22***	(0.07)
Other offenses	0.30***	0.29***	(0.24***)	0.22***	(0.13***)	0.14***	(0.03)

YPI-S = Youth Psychopathic Traits Inventory—Short Version; *** *p* < 0.001; ** *p* < 0.01; SDQ = Strengths and Difficulties Questionnaire

#### Age Group and Gender as Moderators

To test whether gender and age group moderates the associations between YPI-S scores and criterion measures, and, thus, whether results should be presented by age group and by gender, multivariate regression analyses were performed (see footnote Table 5 for detailed information). Table 5 demonstrates a small number of significant associations between interaction terms and criterion measures. For these criterion measures additional analysis were performed to test whether the associations between YPI-S variables and criterion measures varied by age group and by gender. All p-values for the ß’s described below are below <0.01 unless otherwise specified (available upon request).

**Table 5:** Interactions between age group and YPI-S scores and between gender and YPI-S scores

	Emotional problems	Conduct Problems	Hyper-activity	Peer Problems	Prosocial behavior	Violent offenses	Property offenses	Drug-related offenses	Threats and Insults	Other offenses
YPI-S total score ^a^	ß	ß	ß	ß	ß	ß	ß	ß	ß	ß
YPI-S x Age group	−0.29**	0.01	0.21	−0.16	−0.09	−0.06	0.30***	0.59***	0.12	0.19
YPI-S x Gender	0.20	0.11	0.25	0.08	−0.05	−0.21	−0.17	−0.33**	−0.44***	0.24
Interpersonal dimension (ID) ^b^										
ID x Age group	−0.18	−0.02	0.01	−0.13	0.02	−0.08	0.33**	0.54***	0.04	0.14
ID x Gender	0.21	0.12	0.15	0.16	−0.19	−0.07	−0.13	−0.37***	0.46***	−0.11
Affective dimension (AD) ^c^										
AD x Age group	−0.34**	−0.07	0.12	−0.01	−0.17	−0.19	0.23	0.35**	0.24	0.17
AD x Gender	0.19	0.04	0.12	0.09	−0.08	−0.16	−0.15	−0.23	−0.26**	−0.25
Behavioral dimension (BD) ^d^										
BD x Age group	−0.15	0.09	0.36***	−0.18	−0.09	−0.02	0.13	0.38***	−0.02	0.07
BD x Gender	−0.02	−0.01	<0.01	−0.02	0.17	−0.21	−0.12	−0.16	−0.16	0.10

^a^ The standardized YPI-S total score, age group, gender, YPI-S total score X age group and YPI-S total score X gender were simultaneously included as independent variables in this analysis; ^b^ All three standardized YPI-S dimensions, age group, gender, ID X age group and ID X gender were simultaneously included as independent variables in this analysis; ^c^ All three standardized YPI-S dimensions, age group, gender, AD X age group and AD X gender were simultaneously included as independent variables in this analysis; ^d^ All three standardized YPI-S dimensions, age group, gender, BD X age group and BD X gender were simultaneously included as independent variable in this analysis; *** *p* < 0.001; ** *p* < 0.01

##### Age Groups

The YPI-S total score was negatively related with emotional problems in older (ß = −0.15) but not in younger adolescents (ß = 0.06, *p* = 0.23). The association between the YPI-S total score and property and drug-related offenses was stronger in older than in younger adolescents (ß’s = 0.43 vs. 0.37 and 0.32 vs. 0.14 respectively). Likewise, the ID was stronger related with property and drug-related offenses in older than in younger adolescents (ß’s = 0.33 vs. 0.21 and 0.25 vs. 0.16 respectively). The AD was only negatively related with emotional problems in older (ß = −0.25) but not in younger adolescents (ß = −0.01, *p* = 0.79). Despite a significant AD X age group interaction (Table 4), the AD was not associated with drug-related offenses in younger (ß = −0.09, *p* = 0.07) and older adolescents (ß = 0.05, *p* = 0.33). The BD was stronger related with hyperactivity in older than in younger adolescents (ß’s = 0.63 vs. 0.38) and was not associated with drug-related offenses in younger or in older adolescents (ß = 0.11, *p* = 0.03; ß = 0.12, *p* = 0.03).

##### Gender

The total YPI-S score was stronger related with drug-related offenses and threats and insults in boys than in girls (ß’s = 0.33 vs. 0.13 and 0.47 vs. 0.27 respectively). Likewise, the ID was stronger related with drug-related offenses and threats and insults in boys than in girls (ß’s = 0.31 vs. 0.13 and 0.38 vs. 0.19). The AD was only related with threats and insults in boys (ß = 0.17) and not in girls (ß = 0.08 *p* = 0.11). Because there were no significant relations between the BD and gender, performing analyses for boys and girls separately was not warranted.

### Criterion Validity: Person-Oriented Approach

To test whether the YPI-S enables the identification of a group of youths scoring high on all YPI-S dimensions and whether these youths differ in expected ways from other youths, a K-means cluster analysis was performed. Based on previous studies (e.g. Christian et al. 1997; Skeem and Cauffman 2003) we expected a cluster of youths scoring high on all three YPI-S dimensions (i.e. a psychopathic-like cluster), a cluster of youths scoring low on all three dimensions (i.e. a control cluster), a cluster of youths scoring high on AD traits but relatively low on the other dimensions (i.e. a callous-unemotional cluster), and a cluster of youths scoring high on BD traits and relatively low on the other two dimensions (i.e. an impulsive cluster).

The K-means four cluster solution was in line with the four clusters we expected (Table 6). Based on the standardized YPI-S dimension scores, 325 participants could be assigned to the control cluster, 223 to the impulsive cluster, 125 to the callous-unemotional (CU) cluster and 95 to the psychopathic-like cluster. One-way analyses of variance with Bonferroni correction for multiple pairwise comparison demonstrated that participants in the psychopathic-like cluster reported significantly more conduct problems and more violent, property, drug-related, threats and insults and other offenses than youths in the three other clusters (Table 6).

**Table 6:** Mean (M) and Standard Deviations (SD) for variables of interest within clusters and differences between clusters

	(1) Control Cluster (*n* = 325)	(2) CU Cluster (*n* = 125)	(3) Impulsive Cluster (*n* = 223)	(4) Psychopathic-like cluster (*n* = 95)	Pairwise group comparison *p* < 0.01
Cluster Solution	Standardized score	Standardized score	Standardized score	Standardized score	
Interpersonal dimension	−0.55	−0.13	0.11	1.77	
Affective dimension	−0.56	1.25	−0.38	1.18	
Behavioral dimension	−0.72	−0.29	0.81	0.94	
Descriptives	Mean (SD)	Mean (SD)	Mean (SD)	Mean (SD)	
YPS-total score	26.72 (3.15)	35.06 (4.08)	34.41 (3.47)	45.57 (5.17)	4 > 1,2,3; 3,2 > 1
Emotional problems	3.25 (2.34)	3.14 (2.33)	3.77 (2.41)	2.78 (2.03)	4 < 3
Conduct problems	1.44 (1.32)	2.02 (1.44)	2.27 (1.52)	3.03 (1.79)	4 > 1,2,3; 3,2 > 1
Hyperactivity	3.77 (2.18)	4.13 (2.20)	5.44 (2.08)	5.35 (2.97)	4 > 1,2; 3 > 1,2
Peer problems	1.55 (1.48)	1.82 (1.40)	1.65 (1.43)	2.10 (1.76)	4 > 1
Prosocial behavior	8.42 (1.37)	7.73 (1.67)	7.88 (1.69)	6.98 (1.97)	4 < 1,2,3; 3,2 < 1
Violent offenses	0.32 (0.57)	0.60 (0.82)	0.61 (0.75)	1.05 (0.99)	4 > 1,2,3; 3,2 > 1
Property offenses	0.76 (1.47)	0.98 (1.88)	1.85 (2.10)	2.99 (3.06)	4 > 1,2,3; 3 > 1,2
Drug-related offenses	0.04 (0.36)	0.02 (0.13)	0.14 (0.40)	0.32 (0.63)	4 > 1,2,3; 3 > 1,2
Threats and insults	0.19 (0.50)	0.45 (0.79)	0.37 (0.64)	1.21 (1.03)	4 > 1,2,3; 3,2 > 1
Other offenses	0.07 (0.26)	0.16 (0.41)	0.09 (0.29)	0.40 (0.57)	4 > 1,2,3

CU = callous-unemotional; YPI-S = Youth Psychopathic Traits Inventory—Short Version

## Discussion

This study examined the factor structure, the internal consistencies and the criterion validity of the YPI-S in a sample of Flemish normal population adolescents. In general, our study supported the YPI’s three factor structure while relevant indices showed that the instrument is internally consistent. In addition, relations between YPI-S total score and dimension scores on the one hand and external criterion measures on the other were generally in line with our predictions. Using a person-oriented approach demonstrated that the YPI-S is also able to identify youths with high scores on all three dimensions, and importantly, that these psychopathic-like juveniles have more conduct problems and committed more offenses than their non-psychopathic-like counterparts. This study replicated and substantially extended the findings of the original YPI-S study (van Baardewijk et al. 2010). The most remarkable findings will be reflected upon below.

Although it is still debated how many factors best represent psychopathy (e.g. Hart et al. 2007), both the original YPI the YPI-S were explicitly developed in line with the three factor model of psychopathy. The CFA fit indices showed that the three factor structure fits acceptably to our data though not as good as to the data presented by the YPI-S developers (*n* = 1,812, CFI = 0.97, RMSEA = 0.044; van Baardewijk et al. 2010). It would have been possible to increase these fit indices in our sample, for example, by removing items from our model that did not strongly load to their respective factor or to include correlated residuals. For two reasons, we did not follow this strategy. First, we simply wanted to test whether we could replicate the three-factor structure of the YPI-S in a different sample than that used in the YPI-S development study. Second, many instruments are currently available to assess psychopathic-like traits in adolescents. Consequently, we did not want to increase the complexity of the field by adding a modified version of the YPI-S.

An examination of internal consistency indicators reveals good reliability for the YPI-S total score and all three YPI-S dimensions. Only the alpha values for the affective dimension (AD) and behavioral dimensions (BD) were just below the recommended criterion value of .70. Yet, other indices that are independent of scale length, such as MIC, may be preferable for evaluating internal consistency, particularly for brief scales (Poythress et al. 2006). The current study’s MIC values clearly indicated that the mean CITC and the MIC for both dimensions were above the recommended criterion value of .70 and between .15 and .50 respectively. The YPI-S, thus, seems to enable a reliable assessment of CU traits (i.e. the AD of the psychopathy construct) in normal population youth. This finding is of particular interest because previous studies using the Antisocial Process Screening Device (APSD) have demonstrated how difficult it is to reliably measure the concept of CU by merely using 6 items (Poythress et al. 2006). In order to overcome this problem, the APSD developers have developed the 24-item Inventory of Callous and Unemotional Traits (e.g. Frick 2004). By reducing the number of YPI items from 15 in the original YPI to 6 in the YPI-S, the YPI developers chose the opposite direction. Remarkably, both the extended instrument (i.e. ICU) and the shortened instrument (i.e. YPI-S) show good internal consistency and may ameliorate the study of CU traits by means of self-report (ICU: Roose et al. 2010; YPI-S: current study).

Associations with a variety of criterion variables provided evidence supporting the validity for the YPI-S. In line with our expectations, the YPI-S total score was positively and significantly related to externalizing problems, problems with peers and all types of offending. Also, the relations between the YPI-S and criterion variables converge with our expectations. That is, the BD is most strongly related with conduct problems and hyperactivity. Only the AD is negatively related with emotional problems while the interpersonal dimension (ID) is related with self-reported offending. Interestingly, our finding that youths in the psychopathic-like cluster had the lowest level of emotional problems supports the widespread notion that psychopaths do not experience normal levels of emotional tension and turmoil (Patrick 1994). The current study demonstrated that of all three YPI-S dimensions, the ID was most strongly positively related with all types of offenses Although interpersonal traits of the original (i.e. 50-item) YPI have demonstrated to be related with offending, this dimension is not typically seen to be more strongly related with criminal behavior than the behavioral dimension (e.g. Andershed et al. 2002; Poythress et al. 2006). A possible explanation for the current finding relates to the development of the YPI-S itself. By removing original YPI items that loaded highly on different dimensions the ID and the BD may show less overlap in the YPI-S (*r* = .36 in the current study) than in the original YPI (e.g. Skeem and Cauffman 2003: *r* = .59). By better grouping the YPI items into unidimensional factors the relation between the ID and criterion measures of interest may become better apparent when using the short version.

The YPI-S generally seems to work equally well in younger and older adolescent and in boys and girls. There were only a small number of interaction effects that suggested that the relation between YPI-S scores and criterion measures may differ by gender and by age. Additional analyses that were performed to better understand the nature of this interactions generally demonstrated that YPI-S scores and criterion measures were significantly related in both age and gender groups, although these relations were stronger in boys (than girls) and older adolescents (than younger adolescents). Yet, these additional analyses also suggested that a relation between a YPI-S score and a small number of criterion measures may only become apparent in boys (e.g. the negative relation between the AD and emotional problems) or in a specific age group (e.g. the positive relation between the ID and hyperactivity). Future studies are warranted to test whether these gender and age group specific findings can be replicated in other samples of youths, and therefore are relevant to take into account when using the YPI-S to study psychopathic-like traits in adolescents.

The results of the current study need to be interpreted in light of several limitations. First, the participants in this study were community youth attending school. Most seriously antisocial and/or mental ill youth may have been absent the day our survey took place or did not cooperate properly resulting in missing data. In order to examine whether the YPI-S is useful in these juveniles, studies in clinical-referred and justice-involved boys and girls are needed. Second, the current study supports the validity of the YPI-S solely as a research instrument when confidentiality is guaranteed. No conclusion can be drawn about the use of the YPI-S as a clinical assessment instrument in settings where anonymity and confidentiality cannot be provided. Third, the correlational design of our study did not allow investigating causal relationships between variables of interest. Fourth, the current study did not include other self-report measures of psychopathic-like traits and therefore could not examine the convergent validity of the YPI-S.

## Appendix

Table 7

**Table 7:** Means and standard deviations for and correlations between YPI-S items

Interpersonal	Mean (SD)	4	5	8	9	14	16	3	6	10	15	17	18	1	2	7	11	12	13
Item 4	1.75 (0.88)	1.00																	
Item 5	2.24 (1.00)	0.44	1.00																
Item 8	1.98 (0.92)	0.19	0.22	1.00															
Item 9	1.63 (0.85)	0.50	0.52	0.33	1.00														
Item 14	1.58 (0.83)	0.64	0.36	0.21	0.51	1.00													
Item 16	1.63 (0.86)	0.20	0.19	0.37	0.30	0.21	1.00												
Affective																			
Item 3	1.78 (0.94)	0.09	0.17	0.14	0.17	0.04	0.19	1.00											
Item 6	1.36 (0.64)	0.15	0.15	0.17	0.27	0.18	0.17	0.15	1.00										
Item 10	1.42 (0.74)	0.07	0.12	0.17	0.15	0.13	0.22	0.39	0.21	1.00									
Item 15	1.81 (1.02)	0.11	0.14	0.10	0.18	0.12	0.17	0.32	0.22	0.19	1.00								
Item 17	1.31 (0.68)	0.08	0.13	0.15	0.15	0.12	0.22	0.32	0.24	0.37	0.22	1.00							
Item 18	1.83 (0.90)	0.14	0.15	0.17	0.16	0.09	0.20	0.18	0.23	0.21	0.30	0.23	1.00						
Behavioral																			
Item 1	1.17 (0.49)	0.16	0.21	0.09	0.23	0.19	0.14	0.06	0.03	0.06	0.12	0.07		1.00					
Item 2	2.18 (0.84)	0.19	0.18	0.09	0.21	0.15	0.15	0.08	0.01	0.08	0.06	0.10		0.16	1.00				
Item 7	2.42 (0.94)	0.14	0.18	0.03	0.13	0.14	−0.05	−0.01	0.05	−0.04	0.01	0.08		0.10	0.35	1.00			
Item 11	2.72 (0.98)	0.14	0.21	0.11	0.15	0.06	0.14	0.12	0.11	0.11	020	0.12		0.08	0.14	0.18	1.00		
Item 12	2.24 (0.92)	0.22	0.21	0.09	0.22	0.21	0.05	0.02	0.08	−0.01	0.12	0.12		0,17	0.40	0.65	0.27	1.00	
Item 13	1.58 (0.82)	0.22	0.26	0.06	0.24	0.27	0.06	0.05	0.13	0.03	0.10	0.06		0.18	0.18	0.23	0.14	0.30	1.00
